# Colossal Dielectric Constant of Nanocrystalline/Amorphous Homo-Composite BaTiO_3_ Films Deposited via Pulsed Laser Deposition Technique

**DOI:** 10.3390/nano14201677

**Published:** 2024-10-18

**Authors:** Shinya Kondo, Taichi Murakami, Loick Pichon, Joël Leblanc-Lavoie, Takashi Teranishi, Akira Kishimoto, My Ali El Khakani

**Affiliations:** 1 Graduate School of Environmental, Life, Natural Science and Technology, Okayama University, 3-1-1 Tsushima-naka, Kita-ku, Okayama 700-8530, Japan; pa4h17px@s.okayama-u.ac.jp (T.M.); kishim-a@cc.okayama-u.ac.jp (A.K.); 2 Institut National de la Recherche Scientifique (INRS), Centre Énergie, Matériaux et Télécommunications, 1650 Boulevard Lionel-Boulet, Varennes, QC J3X 1P7, Canada; loick.pichon@inrs.ca (L.P.); joel.leblanc-lavoie@inrs.ca (J.L.-L.); 3 Laboratory for Materials and Structures, Tokyo Institute of Technology, 4259 Nagatsuta, Midori-ku, Yokohama 226-8503, Japan

**Keywords:** BaTiO_3_, thin film, colossal dielectric constant, nanocrystalline/amorphous homo-composite, pulsed laser deposition

## Abstract

We report the pulsed laser deposition (PLD) of nanocrystalline/amorphous homo-composite BaTiO_3_ (BTO) films exhibiting an unprecedented combination of a colossal dielectric constant (*ε*_r_) and extremely low dielectric loss (tan *δ*). By varying the substrate deposition temperature (*T*_d_) over a wide range (300–800 °C), we identified *T*_d_ = 550 °C as the optimal temperature for growing BTO films with an *ε*_r_ as high as ~3060 and a tan *δ* as low as 0.04 (at 20 kHz). High-resolution transmission electron microscopy revealed that the PLD-BTO films consist of BTO nanocrystals (~20–30 nm size) embedded within an otherwise amorphous BTO matrix. The impressive dielectric behavior is attributed to the combination of highly crystallized small BTO nanograins, which amplify interfacial polarization, and the surrounding amorphous matrix, which effectively isolates the nanograins from charge carrier transport. Our findings could facilitate the development of next-generation integrated dielectric devices.

## 1. Introduction

Dielectric/ferroelectric materials, such as BaTiO_3_ (BTO), have played a pivotal role in the development of high-density, compact multilayer ceramic capacitors (MLCCs), which are indispensable components of modern electronic devices [1,2]. The volumetric capacitance density of BTO-based MLCCs can be enhanced by reducing the thickness of the dielectric layer or increasing the dielectric constant of the dielectric materials. However, for ferroelectric materials, the relative dielectric constant significantly decreases when the grain size and film thickness of materials like BTO are reduced to <1 μm [3,4,5]. These sizes and scaling effects limit the effectiveness of the aforementioned methods. Another approach to achieving high-density MLCCs is thin film formation. Large dielectric constants, such as those of bulk materials, have been reported in BTO films that are epitaxially grown on single-crystal substrates [6,7,8,9]. The epitaxial mechanical constraint stabilizes distinct domain states, such as ultrafine polymorphic nanodomains, similar to the morphotropic phase boundaries of ferroelectrics, which are absent in bulk form, even within the thin film regime [8,9]. However, only a few studies have reported that polycrystalline films with a thickness of <1 μm exhibited dielectric constants exceeding 2000 [10,11,12,13,14,15,16,17,18,19,20]. Fabricating polycrystalline films with high dielectric constants is challenging, because controlling the mechanical boundary conditions is impractical for these films. A suitable alternative to the highly expensive monocrystalline substrates is polycrystalline films fabricated on affordable and readily available substrates, such as Si wafers or metal sheets, that exhibit colossal dielectric constants (CDCs).

The search for novel materials exhibiting CDC values surpassing those of BTO-based materials has been ongoing in recent decades. Non-ferroelectric materials, such as CaCu_3_Ti_4_O_12_ (CCTO), doped NiO, and TiO_2_-based compounds, exhibit a stepwise dielectric behavior with decreasing temperature and increasing frequency, demonstrating CDCs across a broad temperature range [21,22,23,24,25,26,27,28,29]. The apparent CDC is commonly attributed to Maxwell–Wagner-type extrinsic effects induced by interfacial polarization and formed at the interface between a metal and an insulator or the semiconducting particles surrounded by an insulating interfacial barrier layer, rather than intrinsic dielectric polarization, such as dipole or ionic polarization [30]. However, the materials must suppress dielectric losses while maintaining CDCs, as the hopping carriers within the crystal grains can traverse long distances and easily cross the insulating barrier layer. One approach to controlling dielectric properties is to use heterogeneous materials comprising a conductive or semiconductive core and an insulating shell. Materials such as conductive fillers or nanoparticles embedded in dielectric matrices, and conductive nanomaterials such as Li-ion conductors decorated with an insulating dielectric layer, have been reported [31,32,33,34,35,36]. However, precise control of their dielectric properties and their applications to thin film architecture have not been extensively explored.

This study reports a novel strategy based on the use of the pulsed laser deposition (PLD) technique under vacuum to control the dielectric properties of BTO thin films and investigates the effect of the deposition temperature (*T*_d_) on their structural and dielectric properties. We identified an optimal *T*_d_ of 550 °C that yields BTO films with a CDC exceeding 3000 and a very low dielectric loss (below 5% at 20 kHz). These outstanding dielectric properties were attributed to a self-organized nanocrystalline/amorphous homo-composite structure. 

## 2. Materials and Methods

### 2.1. Film and Electrode Fabrication

**Figure 1:**
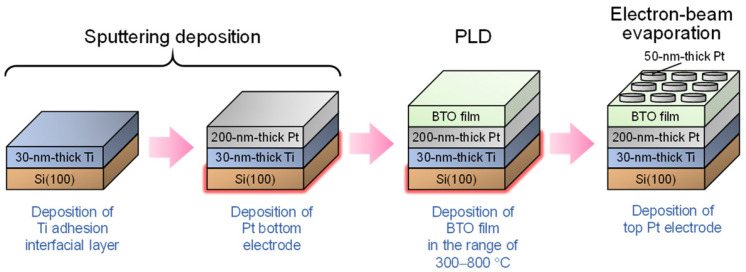
Schematic of the film and electrode fabrication process.

First, the BTO films were deposited on Pt/Ti/SiO_2_/Si (100) substrates using the PLD technique. A bottom ~200 nm thick Pt electrode layer was sputter-deposited with a ~30 nm thick Ti adhesion interfacial layer at substrate temperatures of 450–650 °C by employing an additional bias (−75 V) to achieve highly dense and thermally stable Pt electrodes. The PLD was conducted by focusing a KrF excimer laser beam at an angle of 45° onto a BaTiO_3_ target with a diameter of 2 inches and thickness of 1/4 inches (from Toshima, Japan). The laser pulse energy and repetition rate were set to ~120 mJ and 20 Hz, respectively, resulting in an on-target laser fluence of ~6 J/cm^2^ [37]. The substrates were affixed 7.5 cm parallel to the target surface onto a substrate holder with a diameter of 4 inches. During deposition, the target was rotated at 7 rpm, while the incoming laser beam was swept across its surface to ensure uniform erosion during PLD. The BTO films were grown at various *T*_d_ values in the range of 300–800 °C, while the base pressure in the chamber was maintained at approximately 2 × 10^−5^ Torr. Thus, the PLD was carried out under vacuum without an oxygen background gas. Next, an array of circular Pt top electrodes (50 nm thick with a 100 or 200 μm diameter) were fabricated by depositing Pt through a shadow mask using electron-beam evaporation. The fabrication process of the films and electrodes and their structures are illustrated in Figure 1. The surface of the Pt bottom electrodes was confirmed to be smooth, featureless, and dense prior to BTO deposition, and the interface between the Pt layers and the overlaid BTO film was smooth and distinguishable. This indicates that the Pt layers maintain a highly stable structure and morphology even after BTO deposition at elevated temperatures (Figure S1).

### 2.2. Characterizations of the PLD-BTO Films

The crystalline structure and local bonding states of the PLD-BTO films grown at different *T*_d_ values were determined using X-ray diffraction (XRD) using CuK*α* radiation (Malvern Panalytical, X’Pert, Essex, UK) and Raman spectroscopy with an argon-ion laser at *λ* = 532 nm (Renishaw, Raman spectrometer, Gloucestershire, UK), respectively. The surface morphology and film thickness of the PLD-BTO films were evaluated by field-emission scanning electron microscopy (SEM) (Hitachi, SU-9000, Tokyo, Japan). The dielectric properties of the PLD-BTO films were determined using a precision LCR meter (Keysight, E4980A, Santa Rosa, CA, USA) within the frequency range of 1 kHz to 2 MHz using multiple samples for each *T*_d_. The nanostructure of the PLD-BTO films was studied using a 200 keV Jeol JEM-2100Plus high-resolution transmission electron microscope (HR-TEM). The BTO lamellae for HR-TEM observations were prepared using a high-performance Ga-focused ion beam system (Tescan, LYRA3 XM/GM, Brno, Czech Republic).

## 3. Results

**Figure 2:**
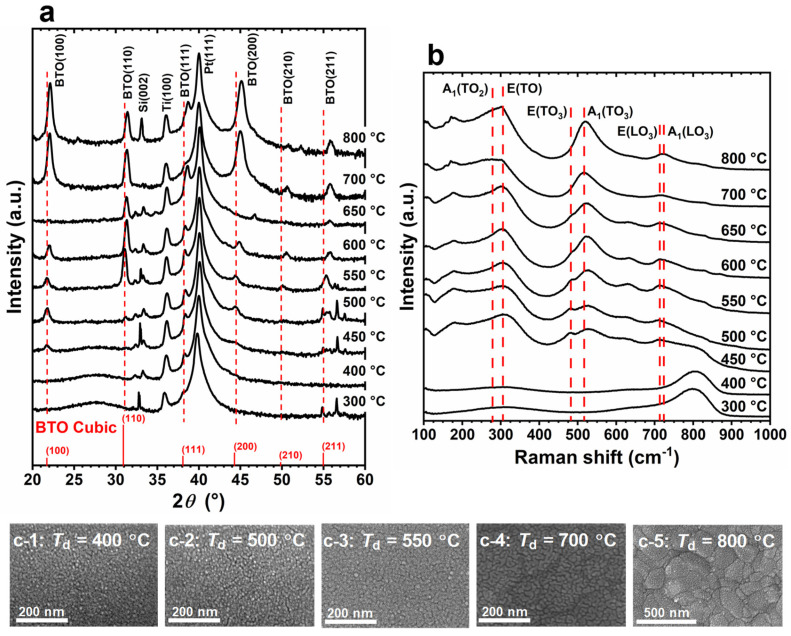
(**a**) Typical XRD 2*θ*/*ω* patterns, (**b**) Raman spectra, and (**c-1**–**c-5**) top scanning electron microscopy (SEM) images of the pulsed laser deposition BaTiO_3_ (PLD-BTO) films grown at different deposition temperatures (*T*_d_) of (**c-1**) 400, (**c-2**) 500, (**c-3**) 550, (**c-4**) 700, and (**c-5**) 800 °C. The cubic BTO XRD peaks are marked in red in (**a**).

Typical 2*θ*/*ω* XRD patterns are shown in Figure 2a. At *T*_d_ ≤ 400 °C, only broad halo peaks were observed at lower diffraction angles, indicating the predominantly amorphous structure of the films. At *T*_d_ ≥ 450 °C, XRD peaks attributed to BTO started to emerge, with their intensity increasing as *T*_d_ increased. This suggests that the crystallinity of the PLD-BTO films increases at higher *T*_d_ values. These results also suggest that crystallization begins at ~450 °C under our PLD conditions. At *T*_d_ = 550 °C, the XRD pattern of the PLD-BTO films is consistent with that of the polycrystalline BTO structure. Notably, the XRD peak positions of BTO are comparable to those of the cubic bulk powder at lower *T*_d_ values (450–550 °C), with no apparent peak splitting. Additionally, the peak positions shifted to higher diffraction angles with increasing *T*_d_ values. These results imply that deposition under vacuum introduces more oxygen vacancies into the films, increasing their lattice volume with increasing *T*_d_ values. The X-ray photoelectron spectroscopy analysis for the PLD-BTO films deposited at different *T*_d_ values supports this presumption. Moreover, the tetragonality of the PLD-BTO films, with a *c*/*a* ratio of nearly unity, indicates an almost pseudo-cubic structure, irrespective of *T*_d_ [38,39,40].

Figure 2b shows the Raman spectra of the PLD-BTO films grown at different *T*_d_ values. The Raman peaks were consistent with those typically observed for BTO films [11,39,41]. However, the Raman peaks became discernible only at *T*_d_ ≥ 450 °C, when crystallization began, which is consistent with the XRD results. Notably, the peak intensity near 512 cm^−1^, attributed to the asymmetric A_1_(TO_3_) modes, increased and narrowed as the *T*_d_ increased. This reflects the enhanced crystallinity of the films, as higher translational symmetry results in strictly phonon-mode oscillation near the center of the Brillouin zone [39]. Moreover, the peak positions of both the A_1_(TO_3_) and A_1_(LO_3_) modes shifted to higher frequencies (blue shift) at a lower *T*_d_ compared to those of bulk BTO [42,43]. This blue shift is attributed to the tensile stress or lattice volume expansion in sub-stoichiometric BaTiO_3−*x*_ films [39,41].

**Figure 3:**
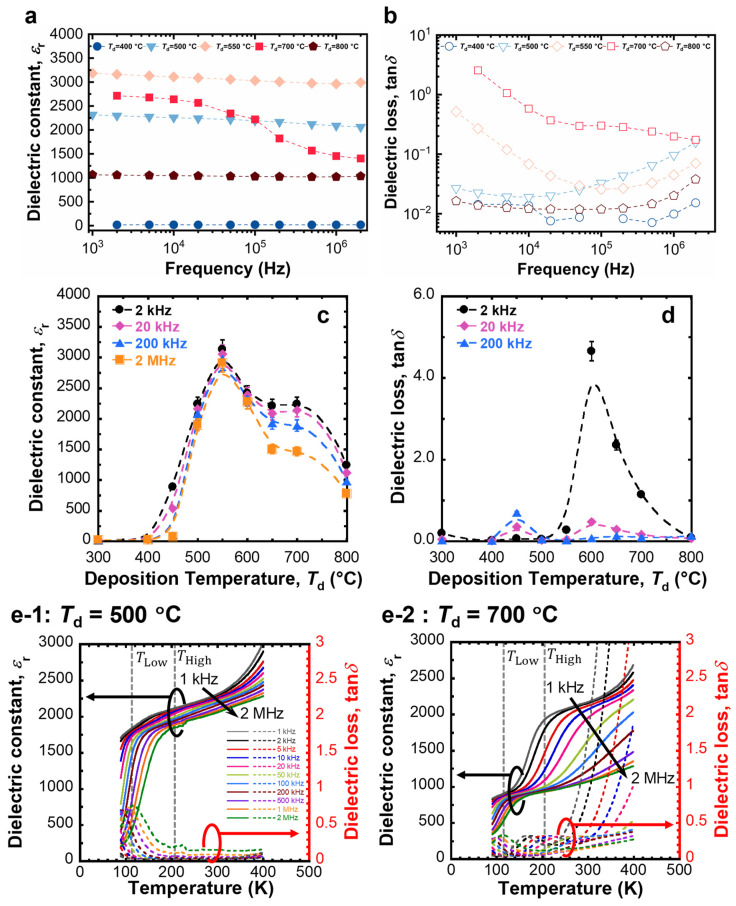
The frequency dependence of the (**a**) dielectric constant (*ε*_r_) and (**b**) dielectric loss (tan *δ*) for films deposited at *T*_d_ = 400, 500, 550, 700, and 800 °C. The *T*_d_ dependence of the (**c**) average *ε*_r_ and (**d**) average tan *δ* at various frequencies. (**e-1**,**e-2**) The measurement temperature dependence of the dielectric properties of the PLD-BTO film deposited at (**e-1**) 500 °C and (**e-2**) 700 °C. The dotted lines in (**a**–**d**) indicate the trend lines.

**Figure 4:**
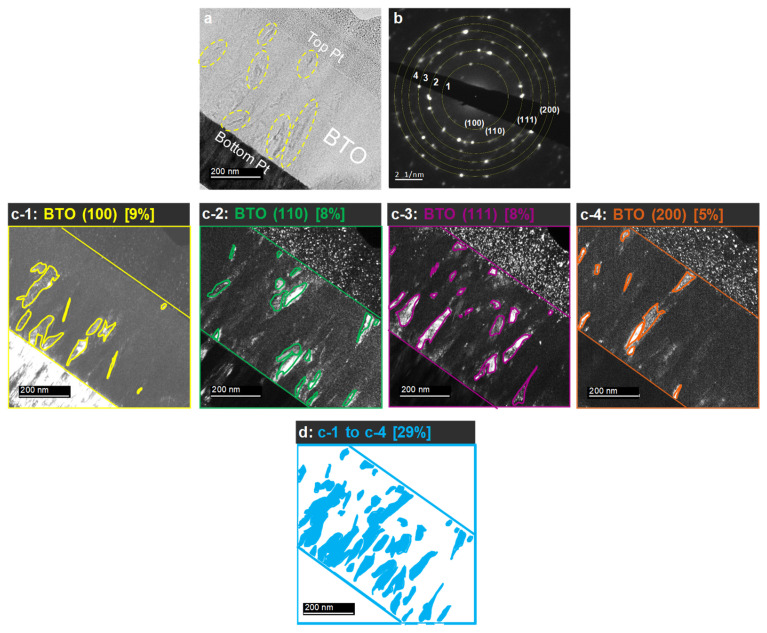
(**a**) A typical HR-TEM cross-sectional bright-field image of the PLD-BTO film deposited at 550 °C. (**b**) The corresponding SAED patterns, showing four facets. (**c-1**–**c-4**) Dark-field images of the (100), (110), (111), and (200) crystal facets on the surface of the BTO film. (**d**) The sum of the fractions of the four BTO nanocrystals facets shown in (**c-1**–**c-4**). This map provides an estimation of the overall crystalline fraction in the PLD-BTO films.

**Figure 5:**
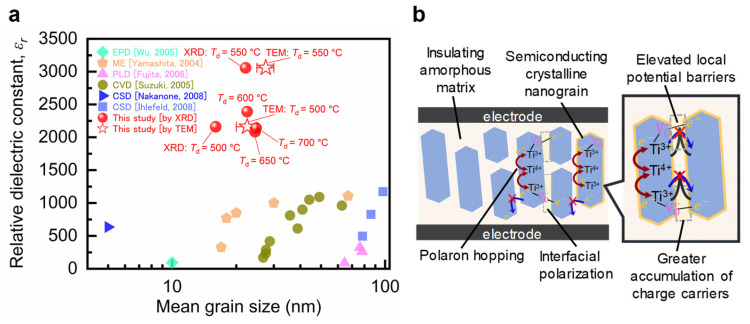
(**a**) The grain size dependence of the dielectric constant of the PLD-BTO films deposited at different *T*_d_ values at 20 kHz and room temperature. The grain sizes of the PLD-BTO films deposited at 500 and 550 °C were calculated from dark-field HR-TEM images. The grain sizes of the PLD-BTO films deposited at other *T*_d_ values were calculated from the XRD-derived crystallite sizes using the Scherrer formula. These dielectric constant values are compared to previously reported values (measured at room temperature in the 1–10 kHz frequency range) for nanograined polycrystalline films prepared by different methods: electrophoretic deposition (EDP) [16], micro-emulsion (ME) [17], PLD [18], chemical vapor deposition (CVD) [19], and chemical solution deposition (CSD) [15,20]. Each color is associated with the references indicated in the figure legend. (**b**) A schematic representation of the nanocrystalline/amorphous homo-composite BTO film illustrating the polarons hopping inside the crystallized grains and their blockage at the grain boundaries because of the presence of the insulating amorphous phase. This blockage leads to the generation of interfacial polarization at the grain boundaries.

Figure 2(c-1–c-5) show the top SEM images of the PLD-BTO films. The BTO granules became coarser as the *T*_d_ increased. The film thickness ranged from 260 nm to 630 nm, depending on the *T*_d_. In the amorphous state (*T*_d_ ≤ 400 °C), the surface of the PLD-BTO films was very smooth and consisted of densely packed granular features with an average size of ~9 nm. As the *T*_d_ increased (up to 700 °C), the granules grew monotonically. At a higher *T*_d_ (≥700 °C), necking was observed between neighboring grains, resulting in further enlargement of the BTO granule size. The average grain size for each *T*_d_ is shown in Figure S2. The average crystallite size, estimated from the BTO (100) and BTO (110) XRD peaks using Scherer’s equation [44], corresponds to the same order observed via SEM (Figure S2).

The typical relative dielectric constant (*ε*_r_) and dielectric loss (tan *δ*) of the PLD-BTO films varied significantly with *T*_d_ (Figure 3a,b). The average *ε*_r_ and tan *δ* for different *T*_d_ values at four frequencies, specifically 2 kHz, 20 kHz, 200 kHz, and 2 MHz, are shown in Figure 3c,d. At *T*_d_ = 400 °C, the films were entirely amorphous, and the *ε*_r_ values remained extremely low, typically in the range of 20–30, characteristic of amorphous ferroelectric films [45,46]. However, beyond the crystallization temperature of the films (*T*_d_ ≥ 450 °C), the *ε*_r_ values significantly increased. The film grown at *T*_d_ = 500 °C demonstrated an *ε*_r_ surpassing 2000, comparable to the *ε*_r_ perpendicular to the polar axis in the bulk single crystal of BTO [47]. Notably, the film grown at *T*_d_ = 550 °C exhibited a maximum *ε*_r_ exceeding 3000, which is comparable to the *ε*_r_ of nanocrystalline bulk ceramics of BTO [3,4]. Despite their overall low crystallinity, the BTO nanograins embedded in an amorphous BTO matrix resulted in a remarkably high *ε*_r_ for the film grown at *T*_d_ = 550 °C. The stable CDC up to the ~1 MHz frequency region cannot be explained by electrode polarization effects due to oxygen vacancy diffusion or complex defect dipoles, such as the ionized oxygen vacancy and Ti^3+^ ion pair, as the relaxation of such polarization typically occurs in the kHz frequency range [46,48,49]. Moreover, the tan *δ* of the films grown at *T*_d_ = 500–550 °C was extremely low. Typically, higher crystallinity and a larger grain size lead to a higher *ε*_r_ [12,13,14,15,17]. In contrast, the *ε*_r_ at *T*_d_ ≥ 600 °C decreased, returning to values in the 750–1000 range at *T*_d_ = 800 °C. For higher *T*_d_ values (≥650 °C), *ε*_r_ decreased significantly with increasing frequencies, and tan *δ* was extremely high, particularly in the low-frequency region. Notably, an abnormally high *ε*_r_ of ~2500 at 10 kHz near room temperature has been reported for low-crystallinity (Ba_0.5_Sr_0.5_)TiO_3_ films prepared using PLD at 550 °C under 1 Pa of N_2_ background gas [50].

To further elucidate the dielectric properties of the films, we investigated the measurement temperature dependence of the dielectric properties of the PLD-BTO films at 80–400 K (Figure 3c). The observed dielectric behavior did not follow the predictions of the Curie–Weiss law as is typical for normal ferroelectric BTO films but rather exhibited a Debye-like thermally excited relaxation process [49]. Therefore, no obvious phase transition was observed in the investigated temperature range. For the low-crystallinity PLD-BTO films grown at *T*_d_ = 500 °C, *ε*_r_ drastically increased at ~100 K, along with a peak in the corresponding tan *δ*, plateaued at 150–330 K, and increased gradually to ≥330 K (Figure 3(e-1)). However, the highly crystalline PLD-BTO films grown at *T*_d_ = 700 °C (Figure 3(e-2)) showed two distinct dielectric loss relaxation points at ~100 (*T*_Low_) and ~200 K (*T*_High_). These features are typically associated with localized carrier hopping within the grain [21,22,23,24,25,26,27,28,29,51,52,53,54,55,56,57]. Moreover, the presence of oxygen vacancies is accompanied by the reduction of some Ti^4+^ ions to Ti^3+^ in the PLD-BTO films [21,53,54,55,56]. Under an applied electric field, the Ti 3*d* electrons in Ti^3+^ ions can hop to their equivalent Ti^4+^ sites, causing lattice distortion [58]. The hopping charge carriers in the lattice create localized hopping dipoles, known as polarons, which in turn generate Maxwell–Wagner-type interfacial polarization [59]. These polarons relax as the sample is cooled. According to the Debye model, a thermally excited relaxation process can be expressed as follows:

(1) $$\tau =\tau_{0}exp\left(-\frac{E_{A}}{k_{B}T}\right)$$

where *τ* is the dielectric relaxation time, *τ*_0_ is the pre-exponential factor, *E*_A_ is the activation energy for relaxation, *k*_B_ is the Boltzmann constant, and *T* is the measurement temperature. The activation energy for each polarization was calculated from an Arrhenius plot using the peak position of tan *δ* according to Equation (1), wherein tan *δ* shifts toward higher temperatures with increasing frequencies (Figure S3). The obtained *E*_A_ values are listed in Table S1. Notably, the *E*_A_ values for *T*_High_ were only slightly larger (by ~0.1 eV) than those of *T*_Low_, irrespective of *T*_d_. The *E*_A_ values for *T*_Low_ were comparable to those reported for polaron relaxation in perovskite oxides (~0.075 eV) [60]. However, the *E*_A_ values for *T*_High_ were comparable to the grain conductivity values, which were generally higher than those for polaron relaxation (0.07–0.15 eV) [51,52,53,61]. Additionally, although the *E*_A_ values at *T*_Low_ and *T*_High_ were comparable, relaxation still occurred at significantly different temperatures. Consequently, the relaxation processes at *T*_Low_ and *T*_High_ were attributed to polaron relaxation and grain conductivity, respectively. The larger sizes of the BTO grains could have led to the large dielectric variation with frequency at *T*_High_ in highly crystalline films [54]. Larger grain sizes lead to longer relaxation times, as charge carriers require more time and energy to drift to the barrier layers (i.e., grain boundaries), rendering it difficult for them to follow the field variations with increasing frequency [34].

Figure 4a shows a typical bright-field image of a cross-sectional view of the PLD-BTO films deposited at *T*_d_ = 550 °C, which exhibits the highest *ε*_r_. The BTO nanograins (yellow circles in Figure 4a) were dispersed in an amorphous matrix. Selected area electron diffraction (SAED) patterns (Figure 4b) showed discrete spots and diffraction rings corresponding to the (100), (110), (111), and (200) facets of the PLD-BTO films, confirming their polycrystalline nature. The low-intensity diffraction spots could be attributed to the dispersion of small BTO nanocrystals in an otherwise amorphous BTO matrix. Subsequently, we selectively irradiated the (100), (110), (111), and (200) crystal facets by centering the objective aperture of the TEM around the corresponding reflection of the SAED pattern. Figure 4(c-1–c-4) show the dark-field images corresponding to the (100), (110), (111), and (200) reflections, respectively. The light contrast reveals the presence of BTO nanograins that exhibit the same crystalline orientation. Regardless of the crystalline orientation, the BTO nanograins were elongated with a width of 20–30 nm and a vertical length of 60–100 nm. Some BTO nanograins coalesced and tended to form BTO “nano-veins” or “nano-filaments” perpendicular to the substrate. To estimate the total volume occupied by the BTO nanocrystals in the PLD-BTO films, the dark-field images of the four different crystalline orientations were summed and plotted in Figure 4d. The total BTO crystalline phase in the PLD-BTO film deposited at *T*_d_ = 550 °C was estimated to be ~29%. Even if this estimation might be conservative, it demonstrates that the films consist of BTO nanocrystals dispersed in an otherwise amorphous BTO matrix, resulting in a BTO homo-nanocomposite film.

The high *ε*_r_ of the homo-nanocomposite, in which highly crystallized domains are dispersed in an otherwise amorphous matrix (with a low *ε*_r_), suggests the occurrence of high polarization in the crystallized regions. Therefore, we interpreted the apparent high *ε*_r_ using a composite model comprising crystal and amorphous phases, applying the Bruggeman and Maxwell–Garnett equations [62]. First, we calculated the dielectric constant of the crystalline portion, *ε*_crystal_, of the PLD-BTO films deposited at *T*_d_ = 550 °C at 20 kHz (*ε*_r_ = 3057 and tan *δ* = 0.04). We assumed the dielectric constant of the amorphous phase to be that of the films deposited at 400 °C (i.e., *ε*_r_ = 29 and tan *δ* = 0.016). This yielded an *ε*_crystal_ of 1–21 × 10^5^ for the volume fractions of the amorphous phase, which ranged from 0.5 to 0.34. These volume fractions are consistent with those derived from the TEM images. These results suggest that a *T*_d_ within the intermediate range of 500–550 °C facilitates crystal nucleation, although complete and uniform crystallization is not achieved. This also indicates that a stabilized nanocrystalline/amorphous composite structure at 500–550 °C stabilizes the *ε*_r_ over a wide range of frequencies and increases interfacial polarization by increasing the interfaces at grain boundaries.

Moreover, the amorphous layer surrounding the BTO nanocrystals may serve as trapping and scattering centers for polarons, impeding their migration between the grains and lowering tan *δ*. Thus, polaron hopping is hindered owing to the elevated local potential barriers at grain boundaries [24]. This confirms that an insulating layer around the grains leads to a greater accumulation of charge carriers at the interface, thereby decreasing the polaron migration between the BTO nanocrystals. The decrease in polaron migration across the grain boundaries results in an increase in grain boundary capacitance and a notable reduction in dielectric losses [52]. This is consistent with previous reports, wherein a crystalline core/amorphous shell structure effectively enhances the resistivity and breakdown strength of CCTO and SrTiO_3_ [63,64,65]. Thus, the application of the nanocrystalline/amorphous homo-composite structure extends beyond BTO and can be implemented in other TiO_2_-based and NiO-based compounds.

## 4. Discussion

Finally, to gain further insights into the grain size dependence of the dielectric constant in nanograined polycrystalline films, we compared our results with the most relevant literature data (Figure 5a). Dielectric constants exceeding 1500 have not been reported for polycrystalline films with particle sizes of <100 nm owing to size and scaling effects [3,4]. Nevertheless, polycrystalline BTO films with grain sizes of >100 nm (approximately five times larger than those of our BTO nanograins) exhibited extremely high dielectric constants, comparable to the dielectric constants found in this study (~3000). The previously reported polycrystalline BTO films were prepared at considerably higher temperatures (>900 °C) to enhance the grain size and achieve a crystalline quality comparable to that of bulk ceramics [13,14]. Our findings confirm that CDCs of >3000 can be achieved in nanocrystalline/amorphous BTO composite films with a BTO grain size of 20–30 nm prepared via PLD at a relatively low thermal budget (*T_d_* = 500–550 °C). A critical factor for the composite to achieve an overall giant dielectric constant is obtaining an “optimal” balance between the size of the highly crystallized BTO grains and the volume of their surrounding amorphous BTO phase. As *T*_d_ increases, the BTO grain size increases; however, the residual amorphous component of the films decreases, thereby reducing the extent of the grain boundaries, which negatively impacts both the dielectric constant and losses. Therefore, we propose that the BTO grains attain the “optimal” size at 550 °C to maximize the polarizability at their interfaces with the surrounding amorphous BTO matrix, while the matrix remains sufficiently “thick” to isolate the highly crystallized nanograins, leading to lower dielectric losses. These results suggest that the effective utilization of the amorphous or low-crystalline phase, which is often considered disadvantageous, can help achieve desirable dielectric properties and represents a paradigm shift in dielectric applications.

## 5. Conclusions

In conclusion, the PLD-BTO films deposited at 550 °C showed a maximum dielectric constant exceeding 3000, coupled with relatively low dielectric loss (approximately 5% at 20 kHz), which is equivalent to those of their bulk ceramic counterparts, despite exhibiting low crystallinity and a small grain size (20–30 nm). Conversely, highly crystalline films prepared above 600 °C exhibited degraded dielectric constants accompanied by high dielectric losses. The PLD-BTO films deposited at 550 °C were confirmed to be self-organized homogeneous nanocrystalline/amorphous composite structures, comprising a semiconducting crystalline core with nanograins and an insulating amorphous matrix. The small size of the BTO nanograins contributes to stable dielectric responses to temperature, frequency, and high interfacial polarization. Moreover, the amorphous interface isolates the nanograins, acting as an effective barrier layer to impede the transport of charge carriers across the grain boundaries (Figure 5b). Our findings highlight the importance of balancing the size of the highly crystallized BTO grains and the volume of the amorphous BTO phase to achieve composite films with unprecedented dielectric properties. Our study also reveals the important role of the amorphous BTO phase in enhancing or tuning the dielectric properties of the BTO homo-nanocomposites at a moderate thermal budget. Furthermore, the compatibility of the PLD technique with advanced microfabrication processing promotes the development of next-generation integrated dielectric devices.

## Data Availability

Data underlying the results presented in this paper are available from the corresponding authors upon reasonable request.

## Supplementary Materials

The following supporting information can be downloaded at https://www.mdpi.com/article/10.3390/nano14201677/s1: Figure S1: SEM images of the top of the Pt bottom electrode prior to BTO deposition and their cross-section after BTO deposition at high temperatures; Figure S2: *T*_d_ dependence of the average grain size estimated from SEM and the average crystallite size estimated from the BTO (100) and BTO (110) XRD peaks of the PLD-BTO films; Figure S3: Arrhenius plot of the frequency dependence of the temperature at which tan *δ* exhibited the maximum of the PLD-BTO films grown at *T*_d_ = 500–700 °C; Table S1: The activation energies of each polarization for the *T*_Low_ and *T*_High_ of the PLD-BTO films grown at *T*_d_ = 500–700 °C.
