# Supplementary material for: Colossal Dielectric Constant of Nanocrystalline/Amorphous Homo-Composite BaTiO3 Films Deposited via Pulsed Laser Deposition Technique
Source: Nanomaterials (Basel). 2024 Oct 18;14(20):1677. doi: 10.3390/nano14201677 (PMC11510155; doi:10.3390/nano14201677)
Supplement: Supplementary file 1 [file nanomaterials-14-01677-s001.zip › nanomaterials-3195193-supplementary.pdf]

Supplementary Information:

Colossal Dielectric Constant of Nanocrystalline/Amorphous Homo-Composite $BaTiO_3$ Films Deposited via Pulsed Laser Deposition Technique

Shinya Kondo[1,*], Taichi Murakami[1], Loick Pichon[2], Joël Leblanc-Lavoie[2], Takashi Teranishi[1,3,*], Akira Kishimoto[1], and My Ali El Khakani[2,*]

[1]*Graduate School of Environmental, Life, Natural Science and Technology, Okayama University, 3-1-1 Tsushima-naka, Kita-ku, Okayama 700-8530, Japan*

[2]*Institut National de la Recherche Scientifique (INRS), Centre Énergie, Matériaux et Télécommunications, 1650 Boulevard Lionel-Boulet, Varennes, QC, J3X 1P7, Canada*

[3]*Laboratory for Materials and Structures, Tokyo Institute of Technology, 4259 Nagatsuta, Midori-ku, Yokohama 226-8503, Japan*

[*]*Corresponding authors: s-kondo@okayama-u.ac.jp; terani-t@cc.okayama-u.ac.jp; m.a.elkhakani@inrs.ca*

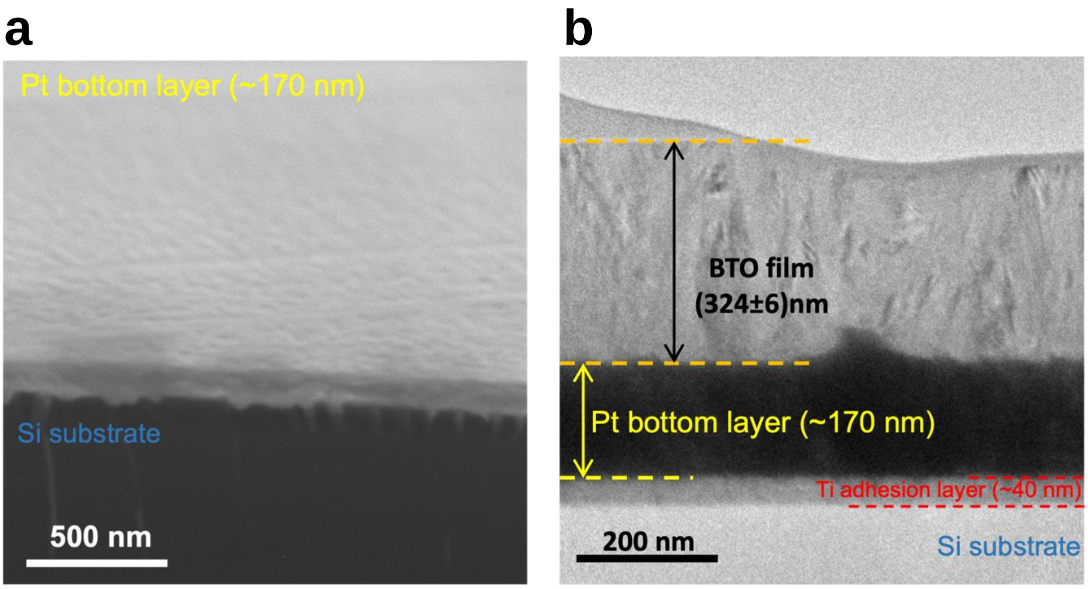

Figure S1. (a) Top SEM image of Pt bottom electrode prior to BTO deposition and (b) their cross-sectional view observed via SEM after BTO deposition at high temperature.

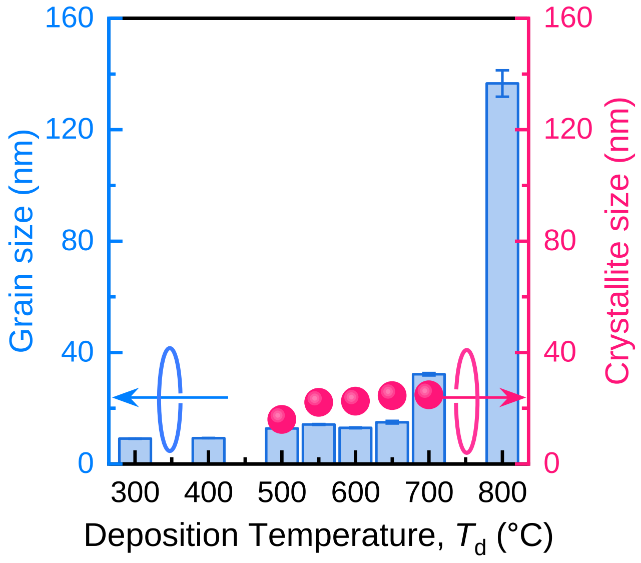

Figure S2. T_d dependence of the average grain size estimated from SEM and the average crystallite size estimated from the BTO(100) and BTO(110) XRD peaks of the PLD-BTO films.

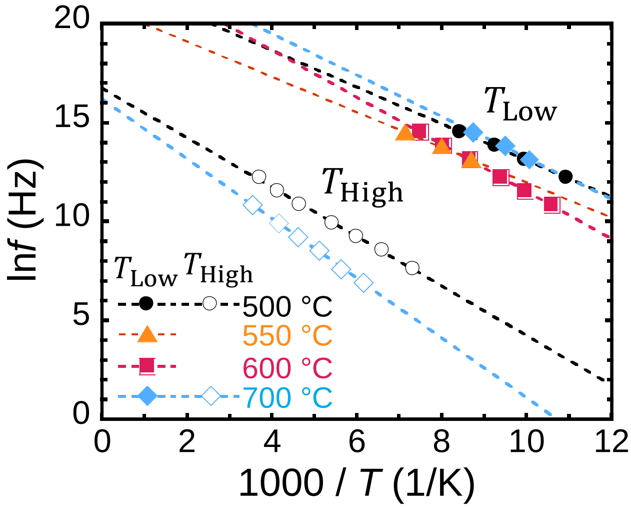

Figure S3. Arrhenius plot of the frequency dependence of the temperature at which tan δ exhibited maximum of the PLD-BTO films grown at T_d = 500–700 °C.

Table S1. The activation energies for each polarization for T_{Low} and T_{High} of the PLD-BTO films grown at T_d = 500–700 °C.

Deposition temperature, T_d (°C)	T_{Low} (eV)	T_{High} (eV)
500	0.08	0.11
550	0.08	–
600	0.10	–
700	0.09	0.13
